# Sparse Ultrasound Imaging via Manifold Low-Rank Approximation and Non-Convex Greedy Pursuit

**DOI:** 10.3390/s18124097

**Published:** 2018-11-23

**Authors:** Thiago Alberto Rigo Passarin, Marcelo Victor Wüst Zibetti, Daniel Rodrigues Pipa

**Affiliations:** Graduate Program in Electrical and Computer Engineering (CPGEI), Federal University of Technology, Paraná (UTFPR), Curitiba PR 80230-901, Brazil; marcelozibetti@utfpr.edu.br (M.V.W.Z.); danielpipa@utfpr.edu.br (D.R.P.)

**Keywords:** ultrasound, nondestructive testing, manifolds, inverse problems, dictionary, rank reduction

## Abstract

Model-based image reconstruction has improved contrast and spatial resolution in imaging applications such as magnetic resonance imaging and emission computed tomography. However, these methods have not succeeded in pulse-echo applications like ultrasound imaging due to the typical assumption of a finite grid of possible scatterer locations in a medium–an assumption that does not reflect the continuous nature of real world objects and creates a problem known as off-grid deviation. To cope with this problem, we present a method of dictionary expansion and constrained reconstruction that approximates the continuous manifold of all possible scatterer locations within a region of interest. The expanded dictionary is created using a highly coherent sampling of the region of interest, followed by a rank reduction procedure. We develop a greedy algorithm, based on the Orthogonal Matching Pursuit, that uses a correlation-based non-convex constraint set that allows for the division of the region of interest into cells of any size. To evaluate the performance of the method, we present results of two-dimensional ultrasound imaging with simulated data in a nondestructive testing application. Our method succeeds in the reconstructions of sparse images from noisy measurements, providing higher accuracy than previous approaches based on regular discrete models.

## 1. Introduction

Model-based image reconstruction methods provided important advances to imaging techniques such as magnetic resonance imaging (MRI) [1] and emission computed tomography (ECT) [2] in the last few decades. These methods rely on a known model that results in the captured signal being represented by a sum of *N* coefficient-weighted responses. These responses are usually point spread functions (PSF), and coefficients are usually intensities of pixels at the modelled locations. Then, by using the discrete model, a vector of acquired data and a regression algorithm, the intensity of each pixel is determined [3]. The use of model-based techniques in ultrasound imaging relies on the assumption that all reflectors (or scatterers) are located on any of a finite grid of *N* modelled positions [4].

Real-world inspected objects easily break this assumption and many scatterers may be situated off-grid. Many previous studies with model-based algorithms for ultrasound imaging [4,5,6,7,8,9,10,11] have reported that resolution and contrast are substantially improved in comparison to delay-and-sum (DAS) algorithms when data comes from simulations with scatterers located strictly on a modelled grid. However, images are corrupted by artifacts when the grid is not respected, which is typical in data acquired from real measurements. Consequently, DAS beamforming algorithms remain as state-of-the-art for ultrasound imaging, despite having well understood physical limitations regarding spatial resolution [12,13].

## 2. Model-Based Imaging and Regularization

**Figure 1:**
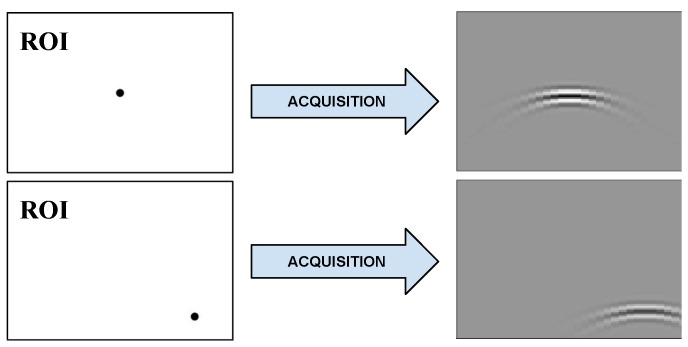
Acquisition of the point spread function (PSF). For each position (x,z) of the unity amplitude scatterer within the region of interest (ROI) (left side), an *M*-sample response $y\left(x,z\right)\in R^{M}$ (arranged as an *M*-pixel image on right side) is generated by the acquisition model. The set of all possible PSFs within the region of interest form a manifold M onto the data space.

Let $R^{M}$ be the space of the data observed through an acquisition process. A single, unity amplitude event located at position $\tau \in R^{D}$ causes the discrete acquired signal $y\left(\tau \right)\in R^{M}$, known as the PSF. In ultrasound imaging, the event denotes a point-like reflexivity (also called a scatterer) [14,15], as represented in Figure 1, and *D* typically equals 2 as the reflexivity is being mapped over a two-dimensional plane. The variation of the set of *D* parameters τ within a region of interest describes a *D*-dimensional manifold

(1) M:={y(τ):τ∈ROI}

of all possible PSFs on $R^{M}$. We will consider the two-dimensional case, where $\tau ={\left[x,z\right]}^{T}$ (being $\cdot^{T}$ the transpose) are the lateral and axial spatial dimensions, respectively.

An acquired signal $c\in R^{M}$ is assumed to be composed by a sum of *N* events, or *N* *samples*, from the continuous PSF manifold

(2) $$c=\sum_{n=1}^{N}v_{n}y\left(x_{n},z_{n}\right)+w,$$

where $v_{n}$ is the amplitude of the *n*-th event and the vector $w\in R^{M}$ accounts for acquisition noise, which we will assume to be Gaussian white noise with variance $\sigma^{2}$.

In a pulse-echo image with *N* pixels, $v_{n}$ in Equation (2) encodes the reflexivity of the *n*-th scatterer, located at position $\left(x_{n},z_{n}\right)$, and is represented as the brightness of the corresponding pixel. This implies sampling the parameters (x,z) on *N* possible scatterer locations (or pixels).

Considering *N* coordinate pairs $\left(x_{n},z_{n}\right)$, we make $h_{n}=y\left(x_{n},z_{n}\right),\;n=1,\cdots ,N$, and define the model matrix $H=\left[h_{1},\cdots ,h_{N}\right]\in R^{M\times N}$. Then, Equation (2) can be written in compact form as

(3) c=Hv+w,

where $v={\left[v_{1},\cdots ,v_{N}\right]}^{T}$ is the vector of scatterer amplitudes. This model has been used in B-mode (two-dimensional) [4,5,6,7,8,9], A-mode (one-dimensional) [16,17], and three-dimensional [18] ultrasound imaging.

The reconstruction of the amplitudes vector v from a given acquisition c in Equation (3) is based on the minimization of a cost function, such as the least squares (LS) problem

(4) $$\hat{v}=\underset{v}{\arg\min}{∥c-Hv∥}_{2}^{2},$$

which is linear and can be solved by well-known methods [19].

However, real-world matrices are often ill-conditioned, which causes artifacts on the reconstructed images in the presence of noise [20], even when all events are on grid. In ultrasound imaging, this problem has been addressed with linear regularization methods such as Truncated SVD (TSVD) [7] and Tikhonov regularizarion [5,6,8].

Sparsity-promoting regularization penalties such as $\ell_{p}$-(pseudo)norm minimization with p≤1 have shown successful results in ultrasound non-destructive testing (NDT), where the assumption of sparsity in the space domain reflects the nature of discontinuities in observed materials [4,9,17,21].

Greedy algorithms effectively solve reconstruction problems where the cost function involves the $\ell_{0}$ pseudonorm. In [10], sparsity is induced in the solution by the assumption that the presence of scatterers can be modelled by a Bernoulli process with a low value for the probability parameter. The problem is then solved with a greedy algorithm called Multiple Most Likely Replacement (MMLR) [22]. In [16], a Gabor dictionary is used in the reconstruction of thickness with a Matching Pursuit (MP)-based algorithm that penalizes a relaxed support measure corresponding to the $\ell_{p}$-pseudonorm with 0<p<1.

## 3. Off-Grid Events and Dictionary Expansion

**Figure 2:**
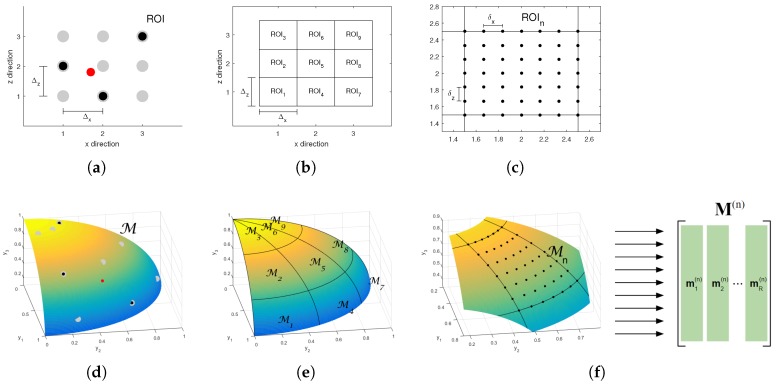
(**a**) an illustrative discrete acquisition model with N=3×3=9 modelled positions, represented by the gray dots. The black dots represent three well located events and the red dot represents an off-grid event. Because of the latter, the corresponding acquisition data vector c cannot be synthesized as a linear combination of the columns of the discrete model matrix H; (**b**) the region of interest (ROI) is divided into *N* local ROIs with area $\Delta_{x}\times \Delta_{z}$; (**c**) each local ROI is sampled with a fine grid with lateral and axial distances $\delta_{x}$ and $\delta_{z}$; (**d**) on the space $R^{M}$ of acquired data, the set of all possible point spread function (PSFs) within the ROI form a manifold M. The gray dots are the PSFs of the modelled positions of Figure 2a. The black dots are on the grid, while the red dot is off-grid; (**e**) as the ROI is divided into *N* local ROIs (Figure 2b), the manifold is divided into *N* corresponding local manifolds; (**f**) the acquisitions over the fine grid on each *n*-th local ROI create *R* samples from the corresponding local manifold. Those samples compose matrix $M^{\left(n\right)}\in R^{M\times R}$.

Aside from poor matrix conditioning, another problem known as off-grid deviation [23] limits the applicability of inverse-problem-based approaches on signal and image reconstruction. Figure 2a illustrates a grid of n=9 modelled positions, represented by gray dots. As three events (represented by black dots) are located on modelled positions, the corresponding data vector c can be synthesized according to the acquisition models of Equations (2) and (3). The same does not hold when an off-grid event (represented by a red dot) is added: attempts to reconstruct the locations and amplitudes for the corresponding events may fail, causing artifacts and degradation on the reconstructed image.

Some formulations have been proposed for off-grid signal reconstruction, mainly within the framework of Compressive Sensing. In [24], the acquisition model considers a perturbation matrix summed column-wise to the (here referred to as H) regular discrete model matrix. The formulation is applied to direction-of-arrival (DOA) estimation using the derivatives of the columns of H with respect to the sampled parameters as perturbation matrix. In [25], an adaptation of the Orthogonal Matching Pursuit (OMP) algorithm is proposed where the columns of the model matrix are iteratively updated in order to accommodate variations in the parameters of the PSFs. The algorithm is applied to pulse-Doppler radar. In [26], the problem of continuous line spectral estimation is approached with an algorithm based on the atomic norm minimization, which is solved via semi-definite programming. Similarly to the $\ell_{1}$ minimization, the atomic norm minimization promotes sparse solutions. In [27], the regression problem uses a Total Least Squares (TLS) penalization with sparsity constraints. The motivation is that the “errors-in-variables” assumption of the TLS regression might be able to capture the mismatch between the model matrix and the acquired data. The method is then applied to cognitive radio sensing and DOA estimation.

Our approach relies on the framework of dictionary expansion. Each column $h_{n}$ of the discrete model H of Equation (3) is replaced by *K* columns $\left[b_{1}^{\left(n\right)},\cdots ,b_{K}^{\left(n\right)}\right]=B^{\left(n\right)}\in R^{\left(M\times K\right)}$ so that a data vector c resulting from the acquisition of an event located in the neighborhood of an *n*-th modelled position can be approximated by some linear combination of $B^{\left(n\right)}$, i.e., by $B^{\left(n\right)}x^{\left(n\right)}$, where $x^{\left(n\right)}\in R^{K}$. As a result, an arbitrarily acquired c might be approximated as

(5) $$c\approx \sum_{n=1}^{N}B^{\left(n\right)}x^{\left(n\right)}.$$

In the two-dimensional case, the neighborhood of the *n*-position is the region within $\left(x_{n}\pm 0.5\Delta_{x},z_{n}\pm 0.5\Delta_{z}\right)$. This is represented in Figure 2b, where the nine modelled locations give place to nine neighborhoods (local ROIs).

Two forms of approximation are proposed in [23] for one-dimensional linear time-invariant (LTI) problems. The first one is the Taylor approximation, which relies on the fact that small shifts on a waveform can be well approximated by its Taylor expansion, i.e., by linearly combining the original waveform and its time derivatives. In this case, the column $b_{1}^{\left(n\right)}$ is identical to the original atom $h_{n}$ and the columns $b_{k}^{\left(n\right)}$ for k>1 correspond to its (k−1)-th time derivatives. The second is the Polar approximation, which is motivated by the fact that the continuous manifold M of an LTI system lies over a hypersphere on the *M*-dimensional data space [23]. The PSFs of the neighborhood of each *n*-th modelled position are approximated by an arc of a circle and the the column $h_{n}$ is replaced by three normal vectors with the directions of the center ($b_{1}^{\left(n\right)}$) and the two trigonometric components ($b_{2}^{\left(n\right)}$ and $b_{3}^{\left(n\right)}$) of the circle. While the Taylor approximation can be done for any order *K*, in the Polar case *K* always equals 3.

An extension of the Basis Pursuit (BP) formulation [28], referred to as Continuous Basis Pursuit (CBP), is proposed in [23] for the recovery of the expanded coefficients ${\left\{x^{\left(n\right)}\right\}}_{1\leq n\leq N}$. For the sake of conciseness, from this point on, we will represent sets ${\left\{x^{\left(n\right)}\right\}}_{1\leq n\leq N}$ simply as $\left\{x^{\left(n\right)}\right\}$. The formulation of CBP is given by

(6a) $$\begin{array}{c} \left\{{\hat{x}}^{\left(n\right)}\right\}=\underset{\left\{x^{\left(n\right)}\right\}}{\arg\min}\frac{1}{2\sigma^{2}}∥c-\sum_{n=1}^{N}B^{\left(n\right)}x^{\left(n\right)}{∥}_{2}^{2}+\lambda \sum_{n=1}^{N}\left|x_{1}^{\left(n\right)}\right|, \end{array}$$

(6b) $$\begin{array}{c} \;s.t.\{x^{\left(n\right)}\}\in C, \end{array}$$

where the constraint set C prevents recovered expanded coefficients from having any arbitrary values that do not represent actual PSFs. The definition of the convex set C varies according to the type of approximation used. The $\ell_{1}$ norm of a vector composed by the first element $x_{1}^{\left(n\right)}$ of each *K*-tuple $x^{\left(n\right)}$ is used to obtain sparse solutions.

In [29], a low-rank approximation of the PSFs within the neighborhood of each *n*-th modelled position is performed by means of a Singular Value Decomposition (SVD). The continuous manifold drawn by τ in a local ROI is sampled with a very fine grid of *R* locations, generating *R* columns that form a matrix $M^{\left(n\right)}\in R^{M\times R}$, as represented in Figure 2f. Each matrix $M^{\left(n\right)}$ then undergoes an SVD decomposition and the *K* first left singular vectors compose the corresponding expanded coefficients $B^{\left(n\right)}$ for the *n*-th local ROI.

An adaptation of the OMP [30] algorithm, referred to as Continuous OMP (COMP), is also presented in [29]. It aims at solving the $\ell_{2}-\ell_{0}$ problem

(7a) $$\begin{array}{c} \left\{{\hat{x}}^{\left(n\right)}\right\}=\underset{\left\{x^{\left(n\right)}\right\}}{\arg\min}{∥\left(x_{1}^{\left(1\right)},\cdots ,x_{1}^{\left(N\right)}\right)∥}_{0}, \end{array}$$

(7b) $$\begin{array}{c} \;s.t.\;\left\{\begin{array}{c} ∥c-\sum_{n=1}^{N}B^{\left(n\right)}x^{\left(n\right)}{∥}_{2}^{2}\leq \epsilon \\ \{x^{\left(n\right)}\}\in C \end{array}\right\}, \end{array}$$

where the symbol ${∥\cdot ∥}_{0}$ denotes the $\ell_{0}$ pseudonorm, i.e., the cardinality (number of nonzero elements) of a vector.

In [31], a minimize–maximum (Minimax) formulation is presented for the definition of the expanded set $\left\{B^{\left(n\right)}\right\}$. The resulting approximation minimizes the maximum residual within the representation of each *n*-th local ROI. It is motivated by the assumption that the off-grid deviation from a discrete grid follows a uniform distribution; therefore, the off-grid error should be as constant as possible, not privileging any distance from originally modelled positions.

## 4. Rank-K Approximation of Local Manifolds

Our criterion to determine $B^{\left(n\right)}$ is based on the SVD expansion, which has been proposed for one-dimensional, shift-invariant problems [29]. The extension to *D*-dimensional problems relies mainly on the first step of the process, which is a fine sampling of each local manifold $M_{n}$: here, the regular, fine grid is defined for all *D* dimensions. This extension is facilitated by the fact that the formulation is non-parametric, i.e., the deviation from originally modelled positions is not mapped onto any independent variable and does not play any role on the definition on the bases. On the other hand, in the Taylor, Polar [23] and Minimax [31] expansions, the off-grid deviation is a parameter from which the elements of the expanded dictionary are derived. Consequently, except for the Taylor expansion, their extensions to two or higher dimensions are not promptly defined.

### 4.1. Highly Coherent Discrete Local Manifolds

Figure 2d shows an illustrative example of a *D*-manifold embedded in an *M*-dimensional data space. In this case, D=2 and M=3. The nine *D*-dimensional modelled positions shown in Figure 2a correspond here to nine samples of the *M*-dimensional manifold, as well represented by gray dots in Figure 2d. The red dot corresponds to the data caused by the off-grid reflector from Figure 2a.

Figure 2e shows the same manifold as Figure 2d, but, instead of having *N* modelled positions, it divides the manifold into *N* local manifolds

(8) $$M_{n}:=\left\{y\left(x,z\right):x\in \left[x_{n}-0.5\Delta_{x},x_{n}+0.5\Delta_{x}\right],\;z\in \left[z_{n}-0.5\Delta_{z},z_{n}+0.5\Delta_{z}\right]\right\},$$

which correspond to the *N* local ROIs of Figure 2b.

We start by performing a fine sampling on each local manifold $M_{n}$, as represented in Figure 2f. In practice, this means acquiring the PSF of a set of points from a fine grid of *R* points defined for each local ROI (Figure 2c). The result is a matrix $M^{\left(n\right)}\in R^{M\times R}$, whose columns are local manifold samples. The finer this grid is, the better the continuous local manifold is represented by the discrete dataset $M^{\left(n\right)}$. For simplicity of notation, we keep regular spacing $\delta_{x}$ and $\delta_{z}$ for the lateral and axial directions, respectively. The number of sampled points is $R=R_{x}\times R_{z}$, where $R_{x}$ and $R_{z}$ are the number of locations defined on the lateral and axial directions, respectively. In the example of Figure 2c, $R_{x}=R_{z}=7$, thus R=49.

Our sampling includes the boundaries of the local ROIs. For this reason, the relation between the spacing and the number of locations on the lateral direction is given by

(9) $$\delta_{x}=\frac{\Delta_{x}}{R_{x}-1}$$

and the same holds for the axial direction.

### 4.2. SVD Expansion

For each matrix $M^{\left(n\right)}$, a rank-*K* approximation ${\tilde{M}}^{\left(n\right)}\in R^{M\times R}$ is to be defined and also factorized in the form

(10) $${\tilde{M}}^{\left(n\right)}=B^{\left(n\right)}F^{\left(n\right)},$$

where $B^{\left(n\right)}$ is an orthonormal basis matrix and $F^{\left(n\right)}\in R^{K\times R}$ modulates $B^{\left(n\right)}$ to form ${\tilde{M}}^{\left(n\right)}$. Any approximation creates a residual matrix $R^{\left(n\right)}\in R^{M\times R}$ defined by the difference

(11) $$R^{\left(n\right)}=M^{\left(n\right)}-B^{\left(n\right)}F^{\left(n\right)}.$$

The SVD expansion is defined by the minimization of the Frobenius norm [19] of $R^{\left(n\right)}$:

(12) $${\hat{B}}^{\left(n\right)},{\hat{F}}^{\left(n\right)}=\underset{B^{\left(n\right)},F^{\left(n\right)}}{\arg\min}{∥M^{\left(n\right)}-B^{\left(n\right)}F^{\left(n\right)}∥}_{F}.$$

According to the Eckart–Young theorem, a solution for Equation (12) is achieved by a truncated SVD [32]. Consider the SVD of M

(13) $$M^{\left(n\right)}=U\Sigma V^{T},$$

where $U\in R^{M\times R}$ is the unitary matrix of left singular vectors, $\Sigma \in R^{R\times R}$ is the diagonal matrix of singular values and $V\in R^{N\times R}$ is the unitary matrix of right singular vectors [19]. The rank-*K* SVD truncation is obtained by using only the *K* largest singular values from Σ and the *K* corresponding vectors from U and V. This low rank approximation is given by

(14) $${\tilde{M}}^{\left(n\right)}=\tilde{U}\tilde{\Sigma }{\tilde{V}}^{T},$$

where $\tilde{U}\in R^{M\times K}$, $\tilde{\Sigma }\in R^{K\times K}$ and $\tilde{V}\in R^{R\times K}$.

The *K* columns of $\tilde{U}$ form an orthonormal basis for ${\tilde{M}}^{\left(n\right)}$ and composes the expanded set $B^{\left(n\right)}$, while the product $\tilde{\Sigma }{\tilde{V}}^{T}$ compose the modulating matrix $F^{\left(n\right)}$:

(15a) $$\begin{array}{c} B^{\left(n\right)}=\tilde{U}, \end{array}$$

(15b) $$\begin{array}{c} F^{\left(n\right)}=\tilde{\Sigma }{\tilde{V}}^{T}. \end{array}$$

**Figure 3:**
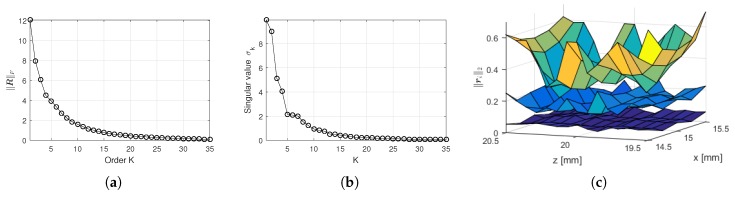
Approximation metrics for the centermost local ROI of the ultrasound acquisition set described in Section 6.1, with R=75 ($R_{x}=5$ and $R_{z}=15$). (**a**) Frobenius norm $∥R^{\left(n\right)}{∥}_{F}$ of the residual matrix as a function of the order of approximation *K*; (**b**) 35 first singular values $\sigma_{k}$ from the singular value decomposition of $M^{\left(n\right)}$; (**c**) individual residual norms $∥r_{i}^{\left(n\right)}{∥}_{2}$ (of columns of $R^{\left(n\right)}$), spatially arranged according to the corresponding positions on the local ROI. The three surfaces correspond to K=5 (top), K=10 (middle) and K=20 (bottom).

Naturally, large values for *K* mean more degrees of freedom in the approximation, which reduces the residuals. Figure 3a shows how the value of *K* affects the Frobenius norm of $R^{\left(n\right)}$ for the centermost local ROI of the acquisition set presented in Section 6.1. The values of the 35 first singular values $\sigma_{k}$ are shown in Figure 3b. The 75 individual residual norms $∥r_{i}∥$ for K=5, 10 and 20 are shown in Figure 3c.

It shall be noted that the processes presented from Equation (12) to Equation (15b) have to be independently performed for every *n*-th local ROI. Although the construction of expanded dictionaries is computationally demanding, it is an offline procedure that is carried only once for each given acquisition set.

## 5. Reconstruction Algorithm

### 5.1. Limitations of Conic Constraints

Two main algorithms were proposed to work with expanded dictionaries: the convex CBP [23] and the greedy COMP [29]. The first one aims at solving the problem of Equation (6) while the second attempts to solve the problem of Equation (7). A hybrid approach called Interpolating Band-excluded Orthogonal Matching Pursuit (IBOMP) was also proposed and applied to frequency estimation (FE) and time delay estimation (TDE) [33]. Basically, it performs a rough greedy estimation of the support of the solution, followed by a refining convex optimization.

**Figure 4:**
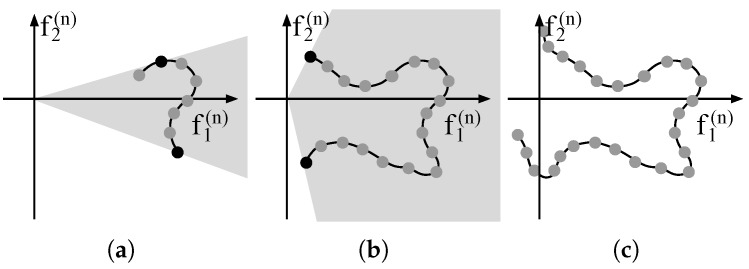
(**a**) illustrative case of projection of local manifold samples $M^{\left(n\right)}$ on a basis $B^{\left(n\right)}$, for K=2. The curved line represents the projection of a continuous one-dimensional manifold, while the dots represent the projection of the samples (columns of $M^{\left(n\right)}$) on $B^{\left(n\right)}$. When Δ is sufficiently small, the projections have single-signed, relatively large values on the first component $f_{1}^{\left(n\right)}$ and smaller values on the remaining components. In this case, the definition of a first-order cone (represented by the shadowed region) is possible and can be used in the reconstruction algorithm combined with a non-negativity constraint for the first component, ensuring that the recovered coefficients represent weighted copies of the local manifold, rather than other arbitrary combinations. The upper and lower angles of the cone depend on $\max_{i}\left(f_{2,i}^{\left(n\right)}/f_{1,i}^{\left(n\right)}\right)$ and $\min_{i}\left(f_{2,i}^{\left(n\right)}/f_{1,i}^{\left(n\right)}\right),$ respectively; (**b**) as Δ increases, the angle of the cone may as well increase, making the constraint less effective, as a broader area is allowed for the recovered coefficients $f^{\left(n\right)}$; (**c**) an example where the definition of a convex cone is no longer possible. This imposes a limit on the definition of Δ.

In order to implement a constraint set C, all the aforementioned algorithms have at least one step involving a constrained convex optimization where the constraints define either first-order (SVD, Minimax and Taylor) or second-order (Polar) cones. Figure 4a illustrates an example of a first-order cone for K=2. The black curved line represents the projection onto the basis $B^{\left(n\right)}$ of a continuous one-dimensional PSF manifold. The *R* vectors that compose a local manifold matrix $M^{\left(n\right)}$, when projected onto $B^{\left(n\right)}$, result in vectors $f^{\left(n\right)}$, represented by the dots, which compose the columns of $F^{\left(n\right)}$. When a reconstruction is performed, the recovered coefficients set $x^{\left(n\right)}\in R^{2}$ for this *n*-th local ROI is constrained to lie within a first-order cone, represented by the shadowed area (which extends indefinitely to the right). This cone is defined by two linear constraints that impose an upper and a lower bound for the relation $x_{2}^{\left(n\right)}/x_{1}^{\left(n\right)}$, combined with a non-negativity constraint for the first component $x_{1}^{\left(n\right)}$. This constraint set aims to avoid arbitrary combinations for $x^{\left(n\right)}$ that do not represent positively-weighted copies of actual manifold samples. The upper black dot defines the upper angle of the cone, and is defined by the modulating matrix $F^{\left(n\right)}$ as $\max_{i}\left(f_{2,i}^{\left(n\right)}/f_{1,i}^{\left(n\right)}\right),$ i.e., the maximum relation between the first and second components found among the projections of $M^{\left(n\right)}$. Similarly, the lower black dot is defined by $\min_{i}\left(f_{2,i}^{\left(n\right)}/f_{1,i}^{\left(n\right)}\right),$ and defines the lower angle of the cone. For higher orders of *K*, such a cone is defined for all K−1 relations between each *k*-th (k≥2) component and the first one. The resulting linear constraint set is defined as [29,31]

(16a) $$\begin{array}{c} \min_{1\leq i\leq R}\left(\frac{f_{k,i}^{\left(n\right)}}{f_{1,i}^{\left(n\right)}}\right)\leq \frac{x_{k}^{\left(n\right)}}{x_{1}^{\left(n\right)}}\leq \max_{1\leq i\leq R}\left(\frac{f_{k,i}^{\left(n\right)}}{f_{1,i}^{\left(n\right)}}\right), \end{array}$$

(16b) $$\begin{array}{c} \;f_{1,i}^{\left(n\right)}\geq 0, \end{array}$$

(16c) $$\begin{array}{c} \;\forall k\in \{2,\cdots ,K\},\;n\in \{1,2,\cdots ,N\}, \end{array}$$

where $f_{k,i}^{\left(n\right)}$ denotes the element on the *k*-th line and *i*-th column on $F^{\left(n\right)}$. The principle is similar for the Polar expansion, though in that case the cones are of second order [23].

Notice that the cone-based convex constraints assume that the projection of $M^{\left(n\right)}$ on the *K* components of $B^{\left(n\right)}$ yields relatively large, positive, small-variance values for the first component and small values for the remaining, yielding relatively small values for minimum and maximum relations of Equation (16c). If this assumption is broken, the cone will span too large an area of the right half-plane, i.e., it will constrain less, being less effective, as represented in Figure 4b. In some cases, defining the the cone is not even possible, as depicted in Figure 4c.

Assuring a well behaved relation between the first and the remaining components, as shown in Figure 4a, implies choosing considerably small values for $\Delta_{x}$ and $\Delta_{z}$, which limits the applicability of recovery algorithms based on conic constraints. For instance, on the simulated acquisition set of Section 6.1, choosing $\Delta_{x}=\Delta_{z}=0.2$ mm still causes the first component to have both positive and negative values on certain local manifolds, which makes the CBP [23], COMP [29] and IBOMP [33] algorithms not applicable.

### 5.2. Non-Convex Constraints

Instead of using conic constraints, our algorithm attempts to constrain each *K*-tuple of recovered coefficients $x^{\left(n\right)}$ to be similar to any column of the modulating matrix $F^{\left(n\right)}$. We translate “similarity” as high correlation, as formalized in the non-convex constraint set

(17) $$\left(\max_{1\leq i\leq R}\frac{\langle x^{\left(n\right)},f_{i}^{\left(n\right)}\rangle }{∥x^{\left(n\right)}∥∥f_{i}^{\left(n\right)}∥}\right)\geq \mu_{c},\;\forall n\in \left\{1,2,\cdots ,N\right\},$$

where $\langle a,b\rangle =a^{T}b$ denotes the inner product of two vectors.

The minimum correlation parameter $\mu_{c}$ controls how similar to any of the manifold samples on $M^{\left(n\right)}$ a recovered event must be. If a given $x^{\left(n\right)}$ passes the test of Equation (17), proving to be sufficiently similar to some *i*-th modulating vector $f_{i}^{\left(n\right)}$, then the approximation

(18) $${\tilde{m}}_{i}^{\left(n\right)}\frac{∥x^{\left(n\right)}∥}{∥f_{i}^{\left(n\right)}∥}=B^{\left(n\right)}f_{i}^{\left(n\right)}\frac{∥x^{\left(n\right)}∥}{∥f_{i}^{\left(n\right)}∥}\approx B^{\left(n\right)}x^{\left(n\right)}$$

is assumed and the product $B^{\left(n\right)}x^{\left(n\right)}$ is considered as a valid weighted copy of a PSF within the *n*-th local ROI, rather than an arbitrary combination of the *n*-th basis vectors. This constraint is imposed by our greedy algorithm on the decision of which expanded set $B^{\left(n\right)}$ will be added to the reconstruction problem at each iteration.

### 5.3. OMP for Expanded Dictionaries

The proposed algorithm, summarized in Algorithm 1, is an extension of the OMP algorithm, referred to as OMP for Expanded Dictionaries (OMPED). It attempts to solve a problem similar to Equation (7) with the non-convex constraint set C defined in Equation (17). The stop criterion is based on the residual yielded by the LS solution with a given cardinality, yet, instead of comparing the residual to a fixed parameter ϵ, we compare it to an estimate of the current residual that takes into account the expected acquisition noise and the estimated residuals resulting from the reduced-rank approximation.
**Algorithm 1** OMP for Expanded Dictionaries (OMPED)**Input:**$\left\{B^{\left(n\right)}\right\}$, $\left\{F^{\left(n\right)}\right\}$, $\left\{R^{\left(n\right)}\right\}$, c, $e_{\mathrm{noise}}$, $\mu_{c}$, $\Delta_{\mu }$1:S←∅2:e←c3:**repeat**4: j← Compute from Equation (21)5: **while** j=∅ **do**
6:  $\mu_{c}\leftarrow \mu_{c}-\Delta_{\mu }$7:  j← Compute from Equation (21)8: **end while**
9: S←S⋃{j}10: $\{x^{\left(n\right)}\}\leftarrow$ Compute from Equation (22b)11: e← Compute from Equation (23)12: $e_{\mathrm{rank}}\leftarrow$ Compute from Equation (24)13: $e_{\mathrm{est}}\leftarrow$ Compute from Equation (25)14:**until**$e_{\mathrm{est}}\geq {∥e∥}_{2}$ or $S^{C}=\emptyset$**Output:***S*, ${\left\{x^{\left(n\right)}\right\}}_{n\in S}$

The input parameter $e_{\mathrm{noise}}$ contains the expected $\ell_{2}$ norm of the acquisition noise. In practice, this value can be obtained from acquisitions with samples of the inspected material known to have neither discontinuities nor other sort of scatterers. For our simulations, we use the relation

(19) $$e_{\mathrm{noise}}^{2}={∥w∥}_{2}^{2}\approx M\sigma^{2},$$

which holds if the noise vector w contains white Gaussian noise with variance $\sigma^{2}$. The approximation of Equation (19) becomes an equality as M→∞. We assume the equality and use $e_{\mathrm{noise}}=\sqrt{M\sigma^{2}}$.

We define the support *S* of the solution, which is initialized as the empty set, and its complement $S^{c}=\left\{1,\cdots ,N\right\}\setminus S$. The solution residual $e\in R^{M}$ is initialized with the vector of acquired data c on line 2.

At each iteration, an index $j\in S^{c}$ is added to *S* as we choose the expanded set $B^{\left(j\right)}$ that is capable of causing the maximal decrease on the energy of the residual, as represented on the left side of Equation (20). Since the columns of each $B^{\left(n\right)}$ are orthonormal, the identity

(20) $$\hat{j}=\underset{j}{\arg\min}∥e-B^{\left(j\right)}{B^{\left(j\right)}}^{T}{e∥}_{2}=\underset{j}{\arg\max}{∥{B^{\left(j\right)}}^{T}e∥}_{2}$$

holds as a consequence of Parseval’s relation [34], which allows us to perform the simpler operation of taking the norm of each product ${B^{\left(j\right)}}^{T}e$.

This operation is a generalization of the measurement of maximum correlation on the original OMP [30]. A constraint based on Equation (17) is imposed to prune candidates that do not accomplish the minimum correlation required. The resulting criterion is formalized as

(21) $$\hat{j}=\underset{j\in S^{C}}{\arg\max}{∥{B^{\left(j\right)}}^{T}e∥}_{2}\;s.t.\;\max_{1\leq i\leq R}\frac{\langle {B^{\left(j\right)}}^{T}e,f_{i}^{\left(j\right)}\rangle }{∥{B^{\left(j\right)}}^{T}e∥∥f_{i}^{\left(j\right)}∥}\geq \mu_{c}.$$

The constraint in Equation (21) allows for the recovery of only positive-amplitude events. It can be adapted to consider both positive and negative amplitudes by simply replacing the inner product by its absolute value $\left|\langle {B^{\left(j\right)}}^{T}e,f_{i}^{\left(j\right)}\rangle \right|$.

The algorithm considers the case where no index meets the correlation criterion of Equation (21). This case is treated from line 5 to line 8: while the problem of Equation (11) remains infeasible, a decrease of $\Delta_{\mu }$ is made on the parameter $\mu_{c}$ and a new attempt to compute the index *j* is performed.

The support *S* is then updated to include the new index *j* (line 9) and is used to compute the coefficients

(22a) $$\begin{array}{c} \left\{{\hat{x}}^{\left(n\right)}\right\}=\underset{\left\{x^{\left(n\right)}\right\}}{\arg\min}{∥c-\sum_{n=1}^{N}B^{\left(n\right)}x^{\left(n\right)}∥}_{2}^{2}, \end{array}$$

(22b) $$\begin{array}{c} s.t.\;x^{\left(n\right)}=0,\;\forall n\in S^{c}, \end{array}$$

where $0\in R^{K}$ is the zero vector. The updated residual is yielded as

(23) $$e=c-\sum_{n\in S}B^{\left(n\right)}x^{\left(n\right)}.$$

Were the manifold approximation exact, e in Equation (23) would be composed strictly of: (1) PSFs located at local ROIs with the corresponding indices not yet added to the support *S* and (2) additive noise. In that case, we could use the widespread stop criterion that compares ${∥e∥}_{2}$ to the expected noise power. However, our residual estimate must take into account the rank-*K* approximation. This estimate is computed on vector $e_{\mathrm{rank}}\in R^{M}$ as

(24a) $$\begin{array}{c} e_{\mathrm{rank}}=\sum_{n\in S}r_{\hat{i}}^{\left(n\right)}\frac{∥x^{\left(n\right)}∥}{∥f_{\hat{i}}^{\left(n\right)}∥}, \end{array}$$

(24b) $$\begin{array}{c} \mathrm{where}\;\hat{i}=\underset{1\leq i\leq R}{\arg\max}\frac{\langle x^{\left(n\right)},f_{i}^{\left(n\right)}\rangle }{∥x^{\left(n\right)}∥∥f_{i}^{\left(n\right)}∥}, \end{array}$$

and $r_{i}^{\left(n\right)}$ denotes the *i*-th column from $R^{\left(n\right)}$. Based on Equation (17), the index *i* in Equation (24b) is a function of *n*: for every index *n* in the current support *S*, the correlations performed in (24b) estimate which *i*-th PSF within the *n*-th local manifold best explains the recovered coefficients $x^{\left(n\right)}$ (see Figure 2c,f). The residual $r_{i}^{\left(n\right)}$, from the dictionary low-rank approximation, is then used as a template for the estimation of the current approximation residual. The amplitude estimate is taken from the ratio between the norms of the recovered coefficients $x^{\left(n\right)}$ and of the similar modulating vector $f_{i}^{\left(n\right)}$.

The current total residual norm is estimated as

(25) $$e_{\mathrm{est}}=(∥e_{\mathrm{rank}}{{∥}_{2}^{2}+e_{\mathrm{noise}}^{2})}^{\frac{1}{2}},$$

where the summation is performed under the assumption that the acquisition noise and the vector $e_{\mathrm{rank}}$ have negligible correlation.

The algorithm greedily increases the support until the estimated residual norm $e_{\mathrm{est}}$ reaches the norm ∥e∥ of the actual residual yielded by the LS or all indices n=1,⋯,N have been added to the support *S*.

### 5.4. Recovery of Locations and Amplitudes

Recalling the approximation $m_{i}^{\left(n\right)}\approx B^{\left(n\right)}f_{i}^{\left(n\right)}$, we determine *i* by finding out which $f_{i}^{\left(n\right)}$ most correlates to $x^{\left(n\right)}$:

(26) $$\hat{i}\left(n\right)=\underset{1\leq i\leq R}{\arg\max}\frac{\langle x^{\left(n\right)},f_{i}^{\left(n\right)}\rangle }{∥x^{\left(n\right)}∥∥f_{i}^{\left(n\right)}∥},\;\forall n\in S.$$

The amplitude estimations $v_{n}$ result form the ratios between the norms of $x^{\left(n\right)}$ and of the chosen template $f_{i}^{\left(n\right)}$:

(27) $$v_{n}=\frac{∥x^{\left(n\right)}∥}{∥f_{i}^{\left(n\right)}∥},\;\forall n\in S,\;i\;\mathrm{as}\;\mathrm{in}\;\text{Equation (26).}$$

As consequence, the spatial resolution of the reconstructed events equals the fine sampling represented in Figure 2c, i.e., $\delta_{x}$ and $\delta_{z}$ for the lateral and axial axes, respectively.

## 6. Empirical Results

### 6.1. Simulated Acquisition Set

**Figure 5:**
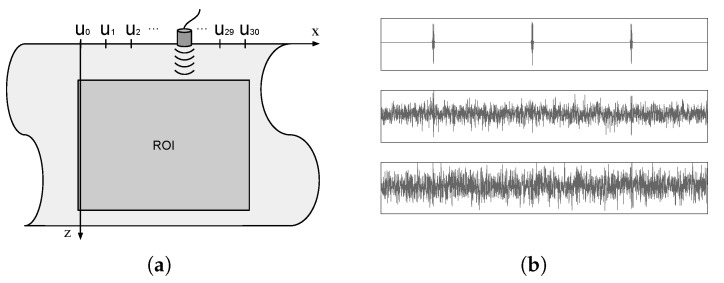
(**a**) simulated set (figure adapted from [21]). The transducer, fixed vertically at z=0, slides horizontally over the surface of the interrogated object, acquiring scanlines at 31 positions $x=\{u_{0},\cdots ,u_{30}\}$, corresponding to 0 mm up to 31 mm with 1 mm step. The scanlines are concatenated to form the acquired vector c. A PSF y(x,z) is determined by placing a unity amplitude scatterer on position (x,z) and acquiring the corresponding c; (**b**) extracts from the acquired data for the three centermost transducer positions, with a unity amplitude scatterer located at the center of the ROI. White Gaussian noise was added with σ=0 (up), σ=0.08 (middle) and σ=0.12 (bottom).

To simulate the ultrasound NDT acquisition set from [21], represented in Figure 5a, we used the Field II package [15] for Matlab^®^ (The MathWorks Inc., Natick, MA, USA). A piston transducer with 3 mm radius (125 μm mathematical element size) interrogates a steel sample object (sound speed c=5680 m/s). The excitation pulse has center frequency $f_{c}=5$ MHz and 6 dB fractional bandwidth of 100%. The simulated transducer slides horizontally along the surface of the object, acquiring scanlines from 31 lateral positions $u_{i}$, from $u_{0}=0$ mm to $u_{30}=30$ mm (center of transducer), with a distance of 1 mm between consecutive lateral positions. The 31 scanlines are sampled with sampling rate $f_{s}=25$ MHz and concatenated to form the acquisition vector c.

Following [21], the model grid has 31×41=1271 modelled locations distributed with regular spacing of 1 mm on both *x*- and *z*-directions. On the *x*-direction, the locations are the same as the transducer positions, i.e., x=0,1mm,⋯,30mm. On the *z*-direction, 41 locations are modelled regularly between 18 mm and 58 mm, i.e., z=18mm,19mm,⋯,58mm.

As explained in Section 4.1, in the expanded acquisition model, the grid locations give place to local ROIs. Our expanded model has 1271 local ROIs with $\Delta_{x}=\Delta_{z}=1$ mm, with centers corresponding to the modelled locations of the regular model. Consequently, our ROI extends from x=−0.5 mm to x=30.5 mm and from z=17.5 mm to z=58.5 mm. The highly coherent local manifolds were created with $R_{x}=5$ and $R_{z}=15$, thus R=75. Therefore, $\delta_{x}=250$μm and $\delta_{z}=71.4$μm.

We simulated the acquisition for 200 cases of five unity amplitude scatterers randomly distributed over the ROI. The scatterers’ positions were not forced over any kind of grid. White Gaussian noise with three different levels (σ=0,0.08,0.12) was added to each simulated acquisition. Since the energy of the acquired signal (without noise) varies according to factors such as distance to transducer and constructive/destructive interference, we consider that the parametrization of noise in terms of its standard deviation σ is more appropriate than signal-to-noise ratio (SNR). To provide a visual notion of the noise levels, Figure 5b shows an extract of acquired data for the three noise levels from an acquisition where a single scatterer was placed on the center of the ROI. Scanlines from the three centermost positions of the tranducer are concatenated.

### 6.2. Recovery Accuracy

**Figure 6:**
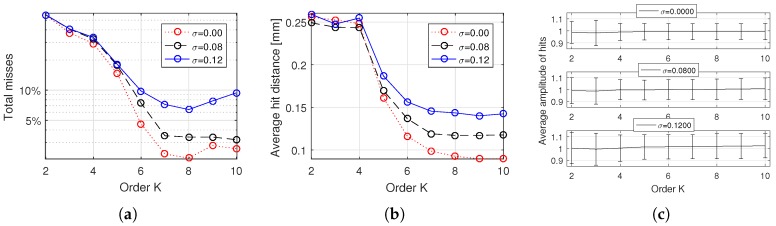
(**a**) percentage of misses (from 1000 simulated events) as a function of K, for three levels of noise, with OMPED running with a fixed number of five iterations (each of the 200 simulated acquisition had five scatterers). Each recovered scatterer distant more than 0.5 mm in any direction (axial or lateral) from the closest original simulated scatterer was computed as a miss. A minimum in the global number of misses is found at K=8. For K>8, a little amount of useful information is added to the dictionary at the expense of increased coherence; (**b**) distance between recovered events (hits) and their corresponding simulated true event; (**c**) average amplitude of the events computed as hits, for noise levels σ=0 (up), σ=0.08 (middle) and σ=0.12 (bottom). The bars indicate one standard deviation above and below the average. All simulated events have unity amplitude.

To compute the accuracy on the recovery of scatterers, we ran OMPED with a fixed number of five iterations, with $\mu_{c}=0.8$, $\Delta_{\mu }=0.1$ and *K* varying from 2 to 10 for the 200 simulated acquisitions with the three levels of noise. Each recovered scatterer distant less than 0.5 mm in both axial and lateral directions from the closest original simulated scatterer was computed as a hit—otherwise, it was computed as a miss. Figure 6a shows the percentage of misses from 1000 recovered scatterers for all nine values of *K* and three noise levels. Even for the highest level of noise, misses kept below 10% for 6≤K≤10. For comparison, we ran OMP with the regular dictionary H on the same set of simulated acquisitions. The resulting percentages of misses were 38.9%, 42.4% and 45.2% for the noise levels σ=0, 0.08 and 0.12, respectively.

A small increase in the count of misses is observed for values of K≥8. This is possibly explained by the fact that, for K≥8, increasing *K* adds few useful information to the dictionary at the cost of increasing coherence. For the SVD basis, the value of the singular values $\sigma_{k}$ can be used as a measure of useful information. Figure 3b shows how $\sigma_{k}$ behaves for the centermost local manifold $M^{\left(636\right)}$. Notice that values of $\sigma_{k}$ for k≥8 are significantly smaller than the previous ones.

For every hit, the distance between the original and the recovered scatterers was computed. The average distances are shown in Figure 6b.

The computation of hits and misses does not take into account the amplitude of recovered scatterers, i.e., recovered scatterers are implicitly considered as having unity amplitude. To endorse this assumption, the average amplitudes of recovered events are shown in Figure 6c, where the bars indicate one standard deviation above and below the average. Notice that, for all cases, the average amplitudes are between 0.98 and 1.01, i.e., the average amplitude error is less than 2%. The average absolute amplitude resulting from the reconstructions with OMP using the regular dictionary H were 0.70, 0.70 and 0.71 for the noise levels σ=0, 0.08 and 0.12, respectively.

### 6.3. Estimation of Residual and Stop Criterion

**Figure 7:**
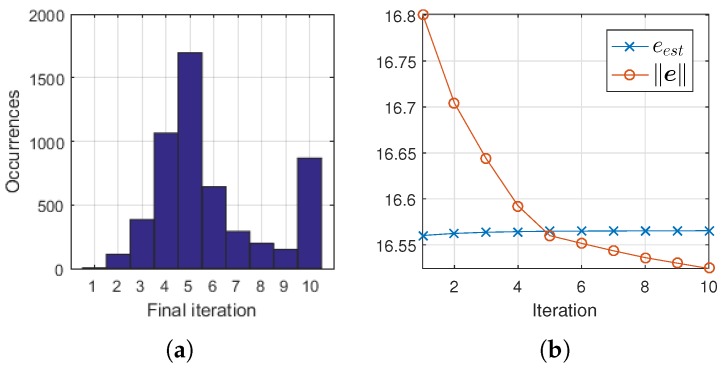
(**a**) histogram of final iteration (when $e_{\mathrm{est}}\geq ∥e∥$ for the first time) for OMPED running with the SVD dictionary, for *K* varying from 2 to 10. Results from all values of *K* are summed. The total number of reconstructions is 5400. The 5th iteration was more frequently identified as final iteration, which is correct since all simulated acquisitions contained 5 scatterers; (**b**) example of evolution of $e_{\mathrm{est}}$ and ∥e∥ along the iterations of OMPED. In this case, $e_{\mathrm{est}}$ dropped below ∥e∥ at the 5th iteration, which was correctly identified as the final iteration. The simulated object contained five scatterers. White Gaussian noise with σ=0.12 was added to the acquired data. OMPED was ran with the SVD dictionary with K=8.

To assess the accuracy of the stop criterion, OMPED was executed one more time on the 5-scatterer dataset of Section 6.1, this time with the residual-based stop criterion defined on line 14 of Algorithm 1, with a maximum of 10 iterations. Because all images contained five scatterers, the algorithm was expected to stop at the 5th iteration. The histogram of Figure 7a shows this outcome: the peak of occurrences is on iteration 5. The frequencies on the neighboring final iterations 4 and 6 are also sensibly greater than on the remaining iterations (except for the maximum 10). The maximum iteration allowed was 10, at which the algorithm stopped when $e_{\mathrm{est}}$ failed to reach ∥e∥. The results for values of *K* from 2 to 10 are summed on the histogram of Figure 7a. A total of 5400 reconstruction (3 noise levels × 200 images × 9 orders *K*) are computed.

Figure 7b shows an example of the evolution of the regression residual norm ∥e∥ and the estimated residual norm $e_{\mathrm{est}}$. As new events are iteratively added to the solution, the latter decreases while the former increases. On iteration 5, ∥e∥ drops below $e_{\mathrm{est}}$ and OMPED correctly meets the stop criterion, yielding a final solution with cardinality 5. White Gaussian noise with σ=0.12 was added to the data. OMPED was ran with SVD (K=8) dictionary.

### 6.4. Reconstructed Images: Examples

**Figure 8:**
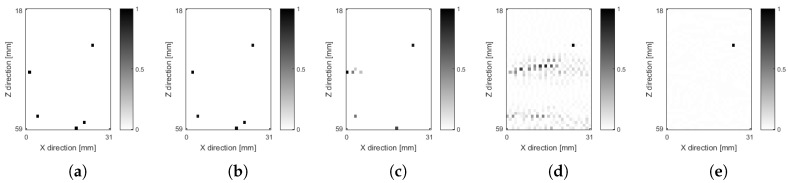
Example of image simulated and reconstructed, from the dataset described in Section 6.1. The simulated data contains five scatterers and white Gaussian noise with σ=0.08. All images are normalized by the maximum absolute pixel value. (**a**) ground truth, with 5 unity amplitude scatterers randomly distributed over the ROI; (**b**) results from OMPED with the SVD dictionary (K=8). The algorithm correctly identified the 5th iteration as the final one; (**c**) results from OMP with regular model H. Seven iterations were run to show that, after the 4th iteration, the algorithm creates artifacts on the neighborhood of the leftmost scatterer instead of identifying the bottom-right scatterer present on the ground truth image. (**d**) Solution of the unregularized LS problem of Equation (4). The image is dominated by artifacts; (**e**) solution of the $\ell_{1}$-regularized problem of Equation (28). The penalization of the recovered amplitudes causes the suppression of most points on the resulting image. The chosen regularization parameter λ=2.0691 minimizes the norm $∥v-\hat{v}∥$, where v is the ground truth.

Figure 8a shows the ground truth for a simulation from the dataset of Section 6.1. Gaussian noise was added to the acquired data with σ=0.08. The reconstructed image using OMPED with SVD dictionary (K=8) is shown in Figure 8b. No limit was imposed on the number of iterations, i.e., the algorithm correctly stopped at the 5th iteration based on the values of the estimated and actual residuals. The activated pixels are the same on the ground truth of Figure 8a and on the OMPED result of Figure 8b. While all simulated scatterers had unity amplitude, the recovered amplitudes ranged from 0.9398 to 1.0387. Both Figure 8a,b have 41×31 pixels corresponding to the local ROIs of the expanded model.

The result of the reconstruction using OMP with the regular dictionary model H is shown in Figure 8c. We ran seven iterations of the algorithm in order to show that, beyond iteration 4, the algorithm created artifacts around the leftmost scatterer instead of identifying the bottom-right scatterer. The recovered amplitudes also display small and even negative values (the image shows absolute, normalized values). Moreover, the bottom-left scatterer is displaced one pixel to the left on the reconstructed image.

Figure 8d shows the image yielded by the LS (unregularized) solution of Equation (4). As is common in unregularized model-based solutions, the image is dominated by noise [35]. We also applied $\ell_{1}$ regularization to the LS problem, which corresponds to the BP formulation [28]

(28) $$\hat{v}=\underset{v}{\arg\min}{∥c-Hv∥}_{2}^{2}+\lambda {∥v∥}_{1}.$$

The $\ell_{1}$-regularized formulation was solved with L1_LS package for Matlab [36]. The resulting image is shown in Figure 8e. While a small value for λ yields an image dominated by noise, such as that of Figure 8d, larger values cause the image to be too sparse, suppressing some features. This is a consequence of the penalization of recovered amplitudes on Equation (28). The chosen regularization parameter λ=2.0691 minimizes the norm $∥v-\hat{v}{∥}_{2}$, where v is the ground truth and $\hat{v}$ is the BP result.

## 7. Conclusions

To cope with the problem of off-grid deviation in image reconstruction from pulse-echo ultrasound data, we developed a technique of dictionary expansion based on a highly coherent sampling of the PSF manifold followed by a rank reduction procedure, as well as a generalization of the OMP algorithm with non-convex constraints. Based on [29], the criterion for the rank reduction is the minimization of the Frobenius norm of the resulting residuals.

Since no assumption is made regarding the geometry of the continuous PSF manifold, our expansion formulation is applicable to both shift-invariant and shift-variant problems. On the other hand, for instance, the Polar expansion [23] is conceived based on the fact that the PSF manifold of any shift-invariant system lies over a hypersphere. In a two-dimensional ultrasound (our main motivating application), the fact that the Spatial Impulse Response (SIR) is spatially variant [15,37] puts the direct acquisition model in the class of shift-variant systems.

The criterion for definition of the order *K* of expansion may vary according to each application. In cases where it is possible to carry out simulations (as presented here) or a relevant amount of data with accessible ground truth is available, *K* can be determined empirically. Moreover, in our case, a minimum in the number of misses is identifiable and lies close to a transition on the baseline of singular values shown in Figure 3b. A suggestion for future studies is the development of a generalized criterion for the definition of *K*. The behavior of the singular values of matrices $M^{\left(n\right)}$ is potentially a starting point for such investigation.

The original OMP algorithm [30] is a particular case of OMPED where K=1 and the parameter $\mu_{c}$ (Equations (17) and (21)) is set to an arbitrarily large negative value. In both OMP and OMPED, the residual vector e on each iteration is orthogonal to all active elements of the dictionary, which places OMPED in the family of *Orthogonal* Matching Pursuit algorithms. The same does not hold for the COMP algorithm presented in [29]: the fact that the LS regression performed at each iteration contains linear constraints may result in eventual coherence between the residual and the active atoms.

Another particularity of OMPED in regard to previously proposed algorithms for expanded dictionaries [23,29,33] is that it is not based on conic constraints, which removes any restrictions on the choice of the sizes $\Delta_{x}$ and $\Delta_{z}$ (and further dimensions if that is the case) for the division of the ROI into local ROIs.

The adaptation of OMP into OMPED, with a constraint imposed on the selection of the index added the support at each iteration, might be replicable to other greedy search algorithms. The class of forward-backward algorithms is of special interest in signal and image recovery because of its capacity of later “correction” of “wrong” choices made on the selection of indices to add to the support [38,39], which constitutes a motivation for future investigation.

The computation of the estimated residual $e_{\mathrm{est}}$ on OMPED may be subject to improvement in order to increase the accuracy of the stop criterion (see Figure 7a). Decreasing the variance of the residuals $r_{i}^{\left(n\right)}$ caused by the low-rank approximation inside each local ROI (i.e., flattening the surfaces of Figure 3c) would cause the inaccuracies on the computation of high resolution locations to have a smaller impact on the computation of $e_{\mathrm{est}}$. This may be achieved with a different criterion for the rank reduction than the LS. For instance, an extension of the Minimax dictionary expansion [31].

One limitation of our technique is that one single point-like event is identifiable inside each local ROI. The search for a means to overcome this limitation, allowing for the recovery of several scatterers inside the same local ROI, is a relevant topic for further investigation and may broaden the applicability of the proposed technique.

Finally, our simulated data considered point-like reflectors, with spatial coordinates (x,z) as the only nonlinear parameters. The ultrasound NDT literature contains parametric reflection models for more complex discontinuity structures, such as spherical voids and circular cracks, where the distortion of ultrasound waves is modelled as a nonlinear function of parameters like diameter and angle to the surface [40,41]. The proposed method is applicable to those cases as long as those parameters are comprised in the parameter set τ in Equation (1) and sampled like the parameters of spatial location. In this case, characterization of discontinuities could be performed along with location. Classification of discontinuities could also be jointly performed if dictionaries for several types of discontinuities are combined. An equivalent principle has been used in the joint detection and identification of neuron activity using SVD [29] and Taylor [42] expanded dictionaries.
